# Simulation-Based Design and Optimization of Accelerometers Subject to High-Temperature and High-Impact Loads

**DOI:** 10.3390/s19173759

**Published:** 2019-08-30

**Authors:** Ji Li, Yaling Tian, Junjie Dan, Zhuming Bi, Jinhui Zheng, Bailin Li

**Affiliations:** 1 College of Mechanical and Electrical Engineering, Chengdu Aeronautic Polytechnic, Chengdu 610100, China; 2 School of Intelligent Manufacturing, Chengdu Technological University, Chengdu 610071, China; 3 Manufacturing Department, Aviation Industry Corporation of China, Ltd., Chengdu Aircraft, Chengdu 610092, China; 4 Department of Civil and Mechanical Engineering, Purdue University Fort Wayne, Fort Wayne, IN 46805, USA; 5 College of Mechanical Engineering, Southwest Jiaotong University, Chengdu 610031, China

**Keywords:** accelerometer, high-temperature high-impact (thermal impact), bolt preload, multi-factor coupling, static analysis, dynamic analysis, virtual experiment

## Abstract

Due to multi-factor coupling behavior, the performance evaluation of an accelerometer subject to high-temperature and high-impact loads poses a significant challenge during its design phase. In this paper, the simulation-based method is applied to optimize the design of the accelerometer. The proposed method can reduce the uncertainties and improve the fidelity of the simulation in the sense that (i) the preloading conditions of fasteners are taken into consideration and modeled in static analysis; (ii) all types of loadings, including bolt preloads, thermal loads, and impact loads, are defined in virtual dynamic prototype of the accelerometer. It is our finding that from static and dynamic analysis, an accelerometer is exposed to the risk of malfunction and even a complete failure if the temperature rises to a certain limit; it has been proved that the thermal properties of sensing components are the most critical factors for an accelerometer to achieve its desired performance. Accordingly, we use a simulation-based method to optimize the thermal expansion coefficient of the sensing element and get the expected design objectives.

## 1. Introduction

### 1.1. Accelerometers in Harsh Environment

System smartness relies on sufficient and reliable data, and this is especially true for intelligent weapon systems. However, it is very challenging to acquire real-time data of motion and impact force with a broad scope of changes in a harsh application environment. Sensing acceleration with a wide range of change is of special interest in this paper, since the information about acceleration subject to high impact is crucial to autonomous control of an intelligent weapon system [1]. When the weapon hits the target, its sensing element serves as an impact switch, while its accuracy is greatly affected by the impact strengths. The sensing element must be designed to be robust to convert the acceleration of the weapon into acceptable signals in the control system, so that the processed data can be used to make correct decisions in a weapon warhead system [2]. In other words, the performance of the sensing system greatly affects the lethality and accuracy of an intelligent weapon system. 

A weapon system must work under extremely hot or cold conditions, such as a mixed condition with high temperature by aerodynamic heating of supersonic flight and a low-temperature environment in high space [3]. The performance of an accelerometer in such a harsh environment can be significantly affected by a wide range of temperature change. When the accelerometer is used as an impact switch, it must function appropriately in the application with high thermal and impact loads.

### 1.2. Sensing Mechanism

As an impact switch of a weapon system, the accelerometer must be designed to be precise and compact for a wide range of measurement. Our research object is a centrally compressed piezoelectric sensor (accelerometer) [4]. An inertial component is a basic sensing element that obtains the impact effect, and then a piezoelectric element is used as a transducer to transfer the impact loads into digital voltage signals. When the piezoelectric element is subjected to stress, the corresponding amount of charge is formed between the surfaces of two polarities. When the stress is removed, the piezoelectric element recovers its electrical neutrality. The piezoelectric element has rapid responsiveness, which allows it to track and obtain instantaneous effects of impact loads at a high sampling rate.

### 1.3. Design Challenges for an Accelerometer Subject to High Temperature and High Impact

To ensure that the weapon system works adequately, the sensing system must sustain the stability of functionalities within the range of measurements subject to various working conditions. As far as the development of a new sensing system is concerned, the system should demonstrate a consistency of performance throughout its product lifecycle from designing, prototyping, manufacturing, transportation, combat application, and maintenance. To take into consideration the effects of impact and temperature loads under different working conditions, the following critical tasks are performed in defining a correct simulation model of the sensing system.

First, an accelerometer in a weapon system must be applicable to the conditions with an impact load up to thousands of gravity acceleration (*g*), and it must be capable of responding to the impact load accurately. This raises a challenge since the sensing element must sustain its structural integrity so that all the required functionalities can be achieved, even in extremely harsh applications. The structure and materials of every part or component must be designed or selected to meet such functional requirements (FRs).

Secondly, the coupled multiphysics behaviors of the sensing element must be taken into consideration since, other than impact loads, a weapon system must withstand a wide range of thermal loads throughout its design and operation cycle. The sensing system is assembled and debugged at the room temperature, stored in the warehouse at around 20 °C for a long time, and deployed in applications at extremely high or low temperature. As the piezoelectric materials possess intrinsic Curie temperature, accelerometers are typically targeted at temperatures of −70 °C, −55 °C, 260 °C, 482 °C, and 648 °C [3,4,5]. 

Accordingly, the sensing system aims for the following FRs:

(1) The sensing system is an assembly of several parts. A failure of any part under an extreme temperature will lead to the failure of the system. Therefore, the materials must be carefully selected so that all the parts are free of failure in the required varying range of temperature. From this perspective, ideal materials should demonstrate the properties of high-temperature resistance such as steel alloys (metals materials) or ceramics (non-metals materials).
(2) The sensing element with the piezoelectric materials must sustain its measuring capability under a high temperature. Many studies have been reported on the development of sensors for applications with high temperature [5].
(3) Taking into consideration a wide and varying range of temperature, the selection of materials should ensure the thermal–structural stability of the sensing system. However, it should be noted that the thermal–structural characteristics of the sensing system are highly coupled with several uncertainties; this presents a difficulty in selecting appropriate materials for parts in the system.
(4) The operation modes reflect different physical conditions for the sensing system. For example, if the system is in the storage, no impact load is involved and the temperature surrounding the system is around 20 °C. It is known as a static state under normal temperature. The conventional combat mode is a combination of the storage mode with an impact load. In addition, both non-combat and combat modes may involve high-temperature conditions. The non-combat mode with a high temperature is the combination of storage mode with a constant high temperature. The combat mode under high temperature is the combination of non-combat mode, high temperature, and impact load. Defining and analyzing the working states is critical to understanding the working mechanism and thus optimizing the design of the sensing system.

### 1.4. The Research Scope of This Paper

In this paper, we aim to develop a robust sensing system to measure the accelerations of a weapon system in any of its deployment conditions. The rest of the paper emphasizes our work in the following aspects: (i) the discussion of sensor structure and the analysis of an experimental scheme; (ii) the development of simulation model; (iii) the analysis of simulation results; and (iv) the use of simulation for design optimization and verification.

## 2. Structural Design and Analysis

### 2.1. Sensor Structure

**Figure 1:**
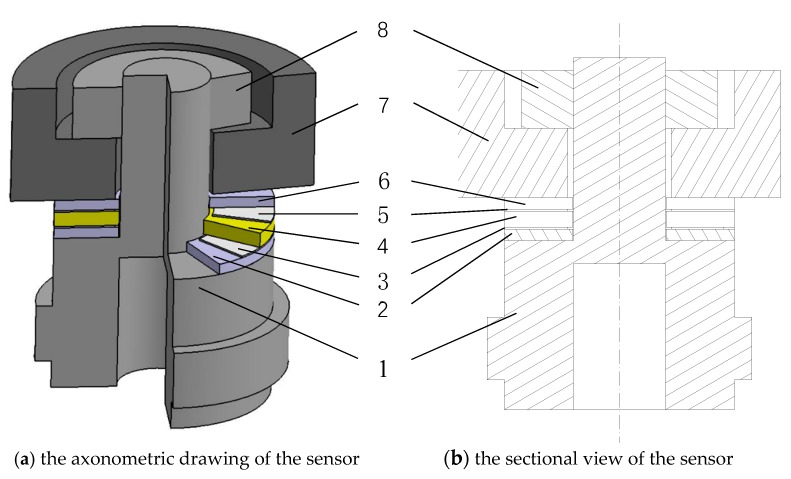
Schematic diagram of sensor structure.

Figure 1 shows the structure of the proposed sensor. As a centrally compressed piezoelectric sensor, it consists of 8 parts, and 2 for the insulating sheet, 3 for the conductive sheet, 4 for the piezoelectric sheet, 5 for the conductive sheet, 6 for insulating sheet, 7 for mass block (inertial component), and 8 for the nuts mounted on the bolt from bottom to top. The entire sensor structure needs a preload which is achieved by initial preloading. Considering all the internal components, compression-type piezoelectric accelerometers are rare [4].

A preload of the bolt causes the piezoelectric sheet to discharge, and thus the sensor has an initial potential, which is also known as the signal baseline. When an impact load is within the working range, the internal structure of the sensing element can generate an elastic oscillation response. Accordingly, the induced stress of the piezoelectric sheet fluctuates, and the output signal from the sensor fluctuates at both positive and negative directions across the signal baseline. Please note that the oscillation of the preload of the bolt is opposite to that of the piezoelectric sheet. The bolt is condensed or elongated in its direction of length with a reference length at the initial preload. If the preload is too low, the oscillation caused by the impact load may introduce an excessive reverse pulling action. This may lead to a case where the contacts within the structure are loosened or even separated with gaps; there will not be any contact stress to generate the piezoelectric signal. On the other hand, if the preload is too high, the oscillation caused by the impact may produce an excessive positive stress superposition and cause the fracture of the sensing element. Therefore, the preload applied to the internal structure of sensor must be traded off. This issue is yet to be discussed in the relevant literature, and thus, the results from existing simulations risk either separation or fracture of sensing elements [6,7]. 

When the sensor is subjected to a thermal load, the thermal adaptation of the overall structure causes a change in internal stress, and it further changes the stress in the piezoelectric sheet. Little literature has investigated the effect of thermal adaptation on the mechanical structures. Zhang mentioned the compensation of the thermal expansion effect for each component by the tightening of nuts and bolts [8]. Cheng mentioned the effect of thermal shock and showed some experimental results, although it is hard to understand the mechanism of the system [9]. The simulation-based method becomes an effective tool for the thermal–structural analysis of the sensing system.

### 2.2. Experimental Scheme

**Figure 2:**
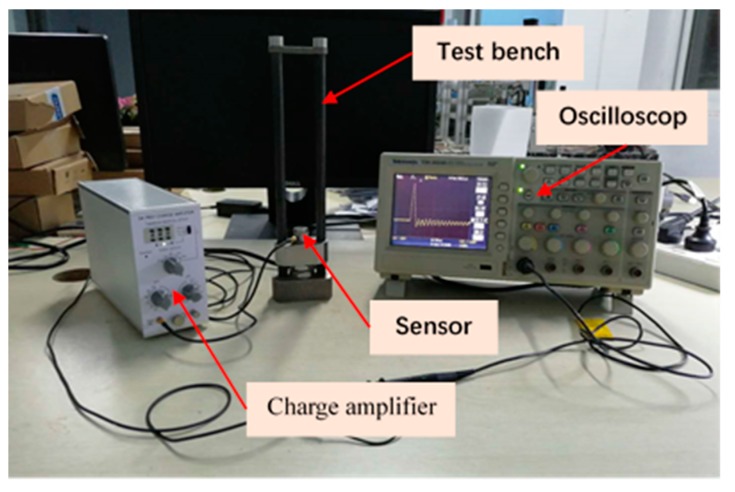
The schematic diagram of the drop test system under normal temperature conditions.

**Figure 3:**
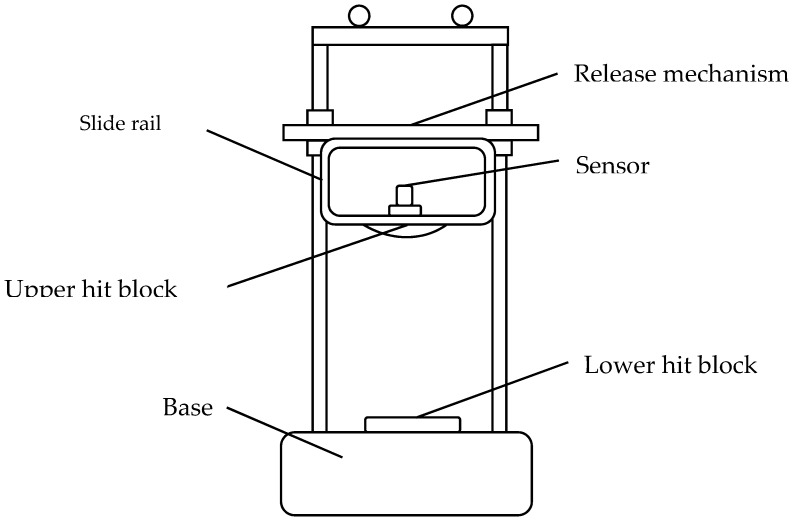
The schematic diagram of the test bench.

Goyal designed a new impact tester and analyzed the multiple impacts of portable electronic products, which demonstrated that the impact direction could be regulated while fulfilling the free-drop conditions [10]. The sensing accelerometer is tested and calibrated by the simple test bench as Liu introduced [11]. Figure 2 shows the test system under normal temperature conditions. Figure 3 shows the principle of drop test. The suitable measuring range of the sensor is 2500 g to 20,000 g, with initial preload of 185 MPa, which is 27% of the bolt material yield limit (685 MPa).

The test bench consists of a base, a slide rail, an upper hit block, a lower hit block, and a release mechanism. The sensing element is mounted on the upper hit block, and the lower hit block is installed on the base. When the upper block is released at a certain height, the sensing element will drop together with the upper hit block, and it collides with the lower hit block with an impact action. The impact action is sensed to generate the piezoelectric signal. The sensing system can clean, filter, amplify, and output the analog signal and display it by an oscilloscope. The signal can be further processed digitally by the software to obtain better waveforms to evaluate the performance of the sensing system. The impact strength can be adjusted by changing the release height. Impact waveforms vary when different shapes of hit blocks are used. For example, we assume that the release height remains the same, the contact time of “flat-head to ball-head” is longer than that of “flat-head to flat-head”. In the “flat-head to flat-head” situation, the contact deformation occurs in the whole plane during the impact process, the normal stress disperses rapidly in the whole plane, so small deformation and short contact time caused by the normal stress. Meanwhile for “flat-head to ball-head”, contact occurs at the point of contact between a plane and a sphere, the force is concentrated, and the elastic deformation occurs near the contact point. The elastic deformation at the contact point further enlarges the contact area until the impact is completely resisted. In this process, the change of the contact area and the contact deformation prolongs the contact time. With such an experimental setup, the amplitude and frequency of the shock wave can be effectively tuned by adjusting the drop height and the shape of the hit block. The uncertainty of the test bench is about 0.048%, which is an effective way to calibrate the inputs and output intuitively and accurately [11,12].

**Figure 4:**
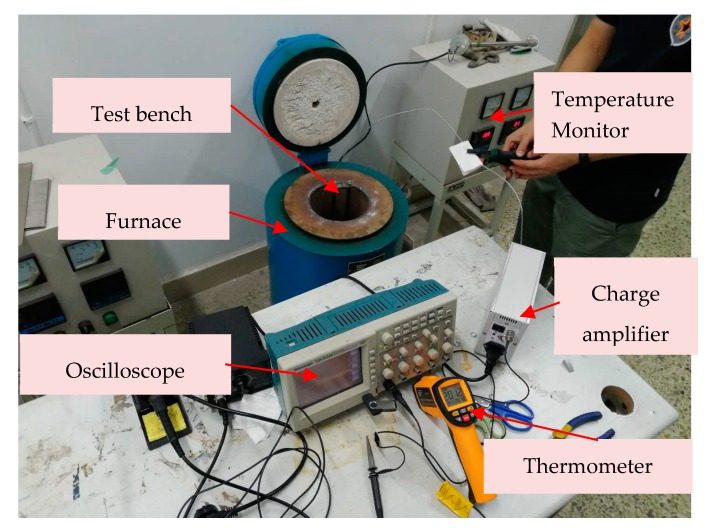
The schematic diagram of the drop test system under 260 °C condition.

Due to its simplicity and effectiveness, the experimental method is widely adopted to characterize the properties of sensing systems. However, the experiments are subject to a high-temperature environment and high-impact loads are very cumbersome. The test bench and signal transmission wires need to be set up in the extra heating furnace; as Figure 4 shows, the test was executed under the temperature of 260 °C.

**Figure 5:**
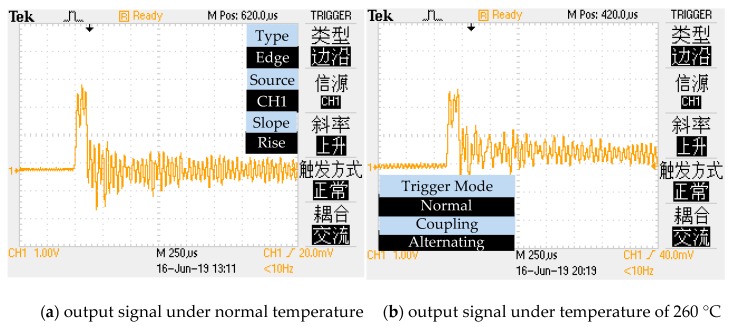
The experimental output signal under normal temperature (**a**) and 260 °C (**b**).

With a drop height of 50-mm under normal temperature and 260 °C, the velocity at the time of collision is 1-m/s, which generates acceleration of 7217 g. We achieved the output signal characteristics as per Figure 5. The fluctuation center is promoted from normal temperature to 260 °C, which means the baseline is rising and the sensor structure is loosening when the temperature rises.

When test temperature is increased to over 300 °C, the soldered dots of the conductor joints and wire coats will melt. The experiments under high temperature and high impact involve a high development cost. It is desirable to use virtual prototyping and simulation in the design and testing of sensing systems.

After reviewing the literature, most of the papers related to the drops are electronic equipment, and no papers were found on the application of numerical simulation of the accelerometer with this test bench [13,14,15,16,17,18,19,20,21,22]. Therefore, this review was extended to consider impact-loading of the whole experimental system, which is the simulation technology of virtual experiment. The virtual experiment is proposed to evaluate the performance of a sensing system, and the proposed virtual simulation aims at several objectives: (i) a simplified and effective simulation model; (ii) the validation and verification of the simulation model by results from physical experiments; (iii) the substitutions of physical experiments in extreme conditions; and (iv) the quantification of thermal and impact loads of mechanical structures. 

## 3. Simulation Model

After reviewing the literature, some papers relate to the simulation of bolt preloads and impacts [23,24,25,26]. Lee simulated a centrally compressed piezoelectric sensor, whose structure is similar to our accelerometer, but only sensitivity and resonance frequency are analyzed [4]. Choi simulated the drop of a haptic actuator, which possesses spring structure, with 2-step drop analysis of external impact and internal impact [27]. No papers were found to model and simulate the coupling of bolt preload, temperature, and impact in dynamic finite-element analysis. 

Without losing generality, the design parameters of a case study sensor in Table 1 are used to illustrate the procedure of developing a simulation model [28,29]. In defining the simulation model, the dynamic and static analyses are combined to take into account of bolt preload effects, temperature changes, and dynamic impact loads. 

Regarding the complexity of coupling conditions, the preloading process is included in the modeling to find the penetration of nut into plate, which is a trial-and-error method to get the required pre-stress [30,31].

### 3.1. Static Analysis with Initial Preloading on Bolt

The commercial software of ANSYS Multiphysics is applied to define, model, and conduct a static analysis.

**Figure 6:**
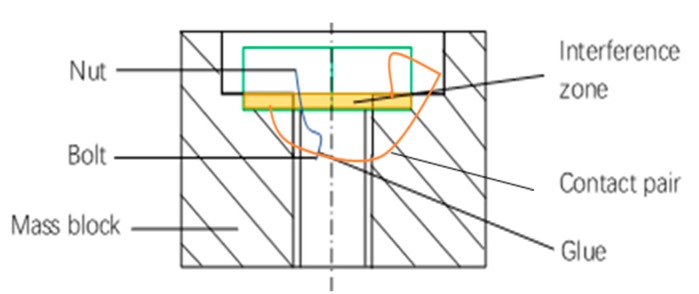
The schematic diagram of nut and mass block with interference.

**Figure 7:**
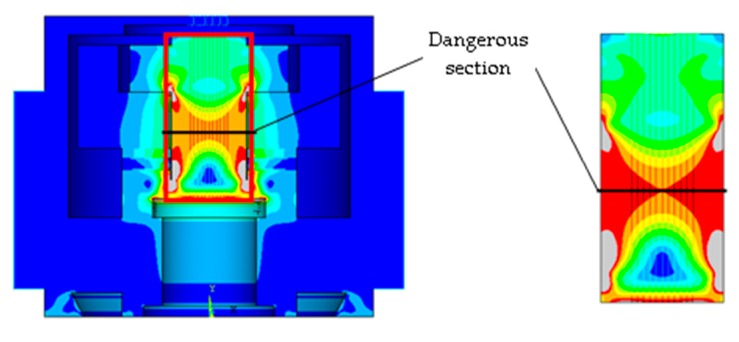
The demonstration of dangerous section.

Step 1-1: The geometries of parts in the sensing systems are modeled by parameters and dimensions in Table 1.
Step 1-2: The initial bolt elongation $\Delta l_{initial}$ is calculated, it is assumed that the connectors are rigid. Based on the given design parameters in Table 2, the tensile stress of the bolt is *P_design_* = 185 MPa, and the initial bolt elongation is determined by
  $$\Delta l_{initial}=\frac{P_{design}L_{0}}{E}$$
  where $L_{0}$ and E are the design length and elastic modulus of the bolt, respectively. Then,
  $$\Delta l_{initial}=6.54\times 10^{-3}\;\mathrm{mm}.$$
Step 1-3: The initial elongation $\Delta l_{initial}$ is treated as an interference amount between the nut and mass block.
Step 1-4: The nut and bolt are set as a glue condition; while other pairs of parts in the model are defined as contact pairs.
Step 1-5: A compulsory contact pair is defined between the lower surface of the nut and the upper surface of the mass block, which is shown in Figure 6.
Step 1-6: The tensile stress of the weakest section of the bolt is analyzed and the result is shown in Figure 7. It is then compared with the design target in Step 2. The nut interference amount must be adjusted until the analysis result is matched with the design value (185 MPa). In this case, the interference amount is obtained as 0.02-mm.

With the static analysis, the initial preloading stress can be expressed accurately with the interference contact preload basing on iterative computation (ICPIC) method [32], and the interference amount will be the input of the dynamic analysis.

### 3.2. Dynamic Modeling and Analysis

The analyses were performed with commercial software of Livermore Software Technology Corporation (LS-DYNA), which is integrated into ANSYS Multiphysics. Using explicit Lagrangian formulations and Temperature-Dependent Bilinear Isotropic (BISO) Material to define and run dynamic simulation models with the following steps.

**Figure 8:**
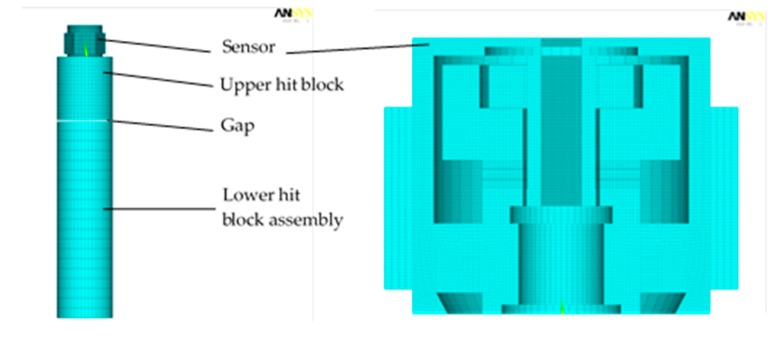
The dynamic simulation for test bench and sensing system.

Step 2-1: The structure of sensor in the test bench (Figure 8) is simplified by suppressing the slide rail. The lower hit block and the base are simplified and glued as a long cylinder (lower hit block assembly). The upper hit block is simplified into a short cylinder of a diameter equal to the lower block. The mass of the short cylinder is set as the same to the mass of the whole free-falling structure in the physical test. In that case, the dynamic model could simulate the same impact of the experiment. The lower surface of the sensor and the upper surface of the upper hit block are glued.
Step 2-2: The collision velocity is calculated. The drop height of 50-mm is commonly used in the physical experiment, accordingly, the velocity at the time of collision is set as v = 1-m/s, which generates acceleration of 7217 g.
Step 2-3: The motion in the physical experiments is simplified by using the principle of relative motions. In the physical experiments, the sensing element and the upper block hit the lower block at the velocity of 1-m/s. To simplify the analysis, the simulation model defines moving objects with a relative velocity and the base part is fixed; i.e., the upper block and sensing element are hit with the constraints of no displacement. The lower hit block assembly moves with the initial velocity of 1-m/s until collide occurs to the upper hit block. The gap between the upper and lower blocks is set as 0.35-mm; this implies that the collision will happen at the time moment of 350-µs. The collision time is thoroughly calculated to adapt the loading of all the simulation parameters. Figure 8 shows the simulation model for dynamic analysis.

### 3.3. Preloading of Bolts in Dynamic Analysis

To investigate the effect of bolt preloading in dynamic analysis, the following steps are involved.

**Figure 9:**
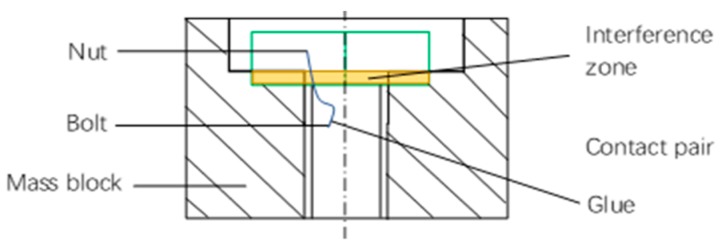
The definition of interference between nut and mass block.

**Figure 10:**
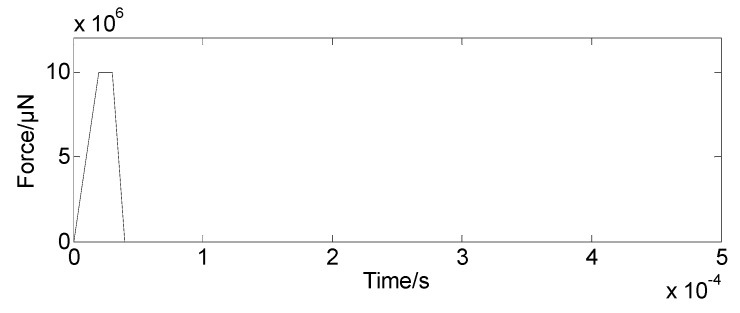
Force with respect to time.

**Figure 11:**
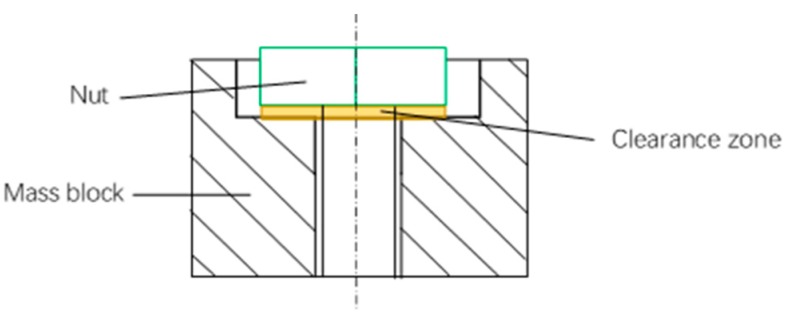
The schematic diagram of nut and mass block with clearance in dynamic analysis.

**Figure 12:**
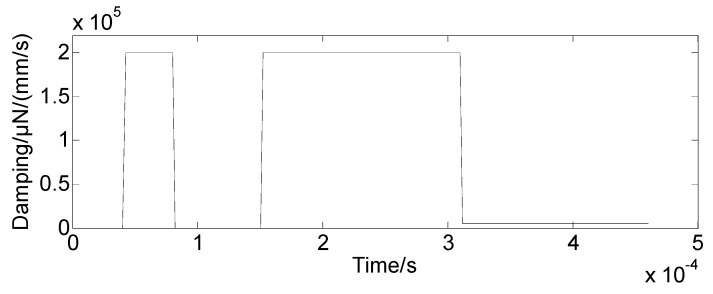
Damping with respect to time.

Step 3-1: The bolt preload interference is defined in the dynamic simulation model, and the value for preloading interference is estimated in Section 3.1. In this simulation, the interference amount is 0.02-mm. In the geometric model, the interference is given between the nut and the mass block; while the nut and bolt are glued. The contact pair of the nut and mass bock is defined at the initial stage and represented in Figure 9. Other contact pairs in the model are defined as face-face contacts.
Step 3-2: The force-time relation is defined, and it is applied on the top end of the bolt to load the preloading action as shown in Figure 10.
  The bolt will be pulled until there is a clearance between nut and mass block. Figure 11 shows such a scenario. Then the pulling force is released to initialize the contacts at the pair of surfaces. The nut will meet and contact with the mass block when the bolt bounces back, and the initial bolt preload is expressed in the dynamic simulation model.
Step 3-3: The dynamic simulation model is an un-damped system, and a sudden change of the load will cause system oscillating. To reduce the computation, it is desirable to shorten the loading time, and it is usually with the units of milliseconds or microseconds. The pulling force should be within the elastic range to avoid the plastic deformation occurring to the bolt. Moreover, the load curve should be as smooth as possible. The model will continue to oscillate after the contact pair is formed. Figure 12 shows the applied damping to eliminate oscillation. The aforementioned methods allow stabilizing the simulation model.

### 3.4. Loading Thermal Load

The thermal loads are taken into account in the dynamic model by the following steps.

**Figure 13:**
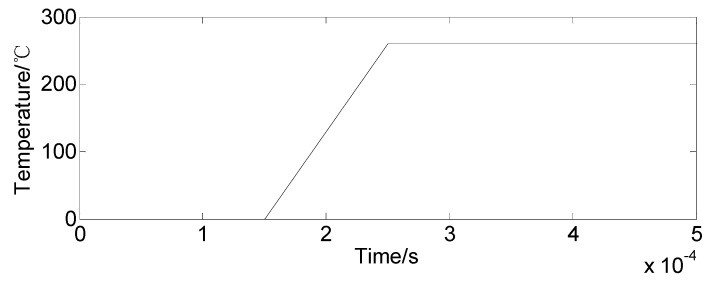
The temperature-time curve.

Step 4-1: The temperature-time curve is defined in the model, and Figure 13 shows the temperature change with respect to time. The thermal load must be within the limit to prevent the sensing system from yielding or damage. The loading time of the temperature change should be extended appropriately, and the temperature-time curve should be as smooth as possible.
Step 4-2: After the thermal loading is completed, the damping is applied to eliminate the oscillation caused by a thermal loading. Finally, a stabilized state is obtained, and the stability state reflects the coupled actions of bolt initial preloading and thermal loads in the simulation model.

### 3.5. Loading Impact

The dynamic impact loads are taken into account in the dynamic model by the following steps.

Step 5-1: Based on the model presented in Section 3.4, the initial velocity is defined for the lower hit block assembly. The impact load is applied by the collision. The sensing system becomes an elastically oscillating system. If the initial velocity is applied on sensing element, the velocity will be built up instantly. The sudden change of the velocity will cause a shock oscillation and an inaccurate reflection of the impact load. If damping is applied to eliminate the shock oscillation, the motion of the sensing element is also restricted, and the collision will be reflected inaccurately. Therefore, it is desirable to set the lower hit block assembly as a moving part.
Step 5-2: After the collision is completed, the sensing system will produce an un-damped periodic oscillation. In fact, the sensing system is a damped elastic oscillation system, and the waveform of the oscillation will be gradually decayed. A certain damping-time curve should be applied to the sensing system to represent the attenuation of the oscillation.

## 4. Analysis of the Simulation Result

### 4.1. The Illustration of the Whole Dynamic Process

**Figure 14:**
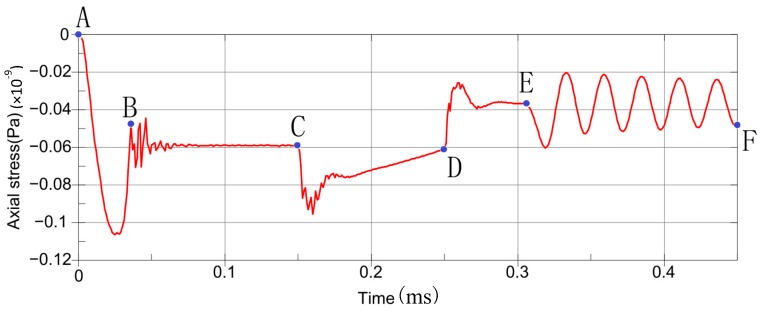
The relation of axial stress-time curves of piezoelectric sheet at 260 °C.

In this section, we focus on the stress of piezoelectric sheet and the stability of the sensing system. The average axial stress of the piezoelectric sheet is calculated to illustrate the dynamic process described in Section 3. The design parameters under the given working conditions are set as 260 °C, the interference amount of 0.02-mm, and the initial velocity of lower hit block 1 m/s (equivalent drop height is 50 mm). Figure 14 shows the analysis results with the following details.

(1) A–B corresponds to the preloading process of bolt.
(2) B–C shows the oscillation produced by preloading has been stabilized by damping. The stress value after the stabilization is −59.1 MPa. This explains the effect of bolt preload.
(3) C–D shows the loading process of thermal loads and the incurred oscillation.
(4) D–E shows the oscillation induced by the thermal loads has been eliminated by damping, and the stress value is −37.1 MPa. That means the sensor structure becomes loose due to the thermal action, which is similar to the characteristics of experimental test. The compressive stress on piezoelectric sheet is decreased by 22.0 MPa.
(5) E–F shows that the impact action and its oscillation have a positive amplitude of −60.8 MPa (compressive stress) and a reverse amplitude of −20.1 MPa (compressive stress). In this situation, the curve reflects the baseline of the sensing element is −37.1 MPa, and it detects the compressive stress of the forward impact strength of −23.7 MPa, and the tensile stress of the reverse impact strength of 17 MPa.

### 4.2. Characteristics of Thermal Structure

**Figure 15:**
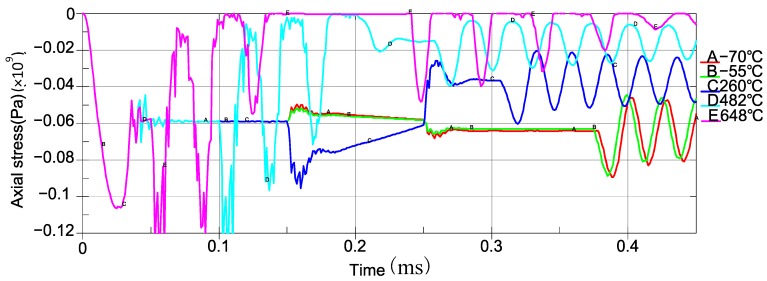
The relation of axial stress with respect to time with different temperatures

Based on the specified FRs, the typical operating temperatures of the sensing systems are −70 °C, −55 °C, 260 °C, 482 °C, and 648 °C. The relation of axial stress with respect to time of piezoelectric sheet at different temperatures are shown in Figure 15. The details of the system responses under different temperatures from the simulation are presented in Table 3.

**Figure 16:**
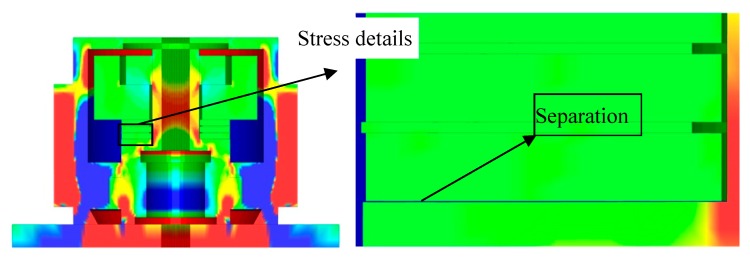
Separation caused by the temperature of 648 °C.

Based on the simulation results, the sensing system with the specified geometry and dimensions of parts and material properties can work appropriately at 260 °C, −55 °C, and −70 °C. However, the baseline fluctuates in a large range (27.7 MPa). The bolt preload effect is relaxed with the thermal action of 482 °C, and the response curve is distorted. While at a temperature of 648 °C, there will be a separation, as Figure 16 shows. The sensing system will fail, and it will not be able to respond to any impact load correspondingly.

It can be seen that the preload of the bolt is weakened when the temperature rises. Therefore, the thermal relaxation is the main factor, and it may cause a failure of the sensing system when a high temperature occurs in the application.

## 5. Design Optimization

Based on the results of above static and dynamic analysis, the following actions are taken to improve the thermal–structural performance of the sensing system.

**Figure 17:**
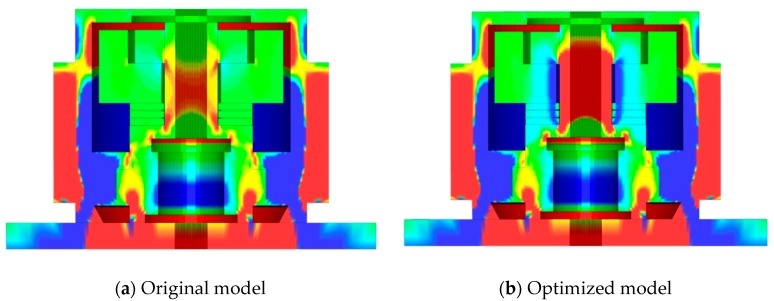
The contrasts of bolt stress caused by temperature of 648 °C.

**Figure 18:**
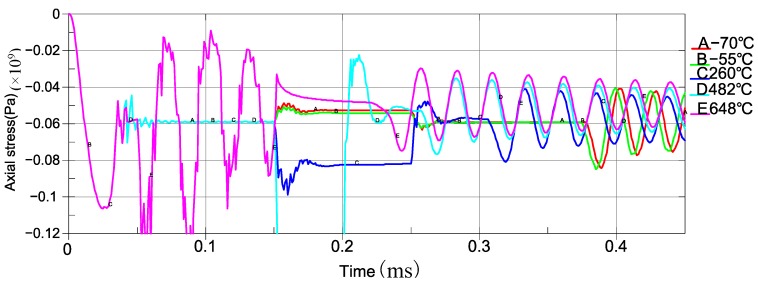
Average axial stress with respect to time under different temperature in optimized design.

(1) Increasing the preload of the bolt. The response baseline of the sensing system can be promoted by increasing the initial preloading stress, which contributes to resisting the reverse impact and increasing the range of thermal load.
(2) Thermal expansion coefficient of the sensing structure should be optimized. According to the analysis result of Section 4.2, the effect of the preload of bolt was relaxed greatly with the increase of temperature, and the sensing system even malfunctioned when the temperature reached 648 °C. Therefore, the thermal expansion coefficient is a key factor to improve the thermal work capacity and stability. We choose this scheme as the benchmarking reference to optimize the sensor design.
(3) The thermal expansion coefficient of mass block is optimized. The mass block does not play a significant role but contributes to the inertial impact; it has the maximized length along the axial direction. Figure 17 shows that the optimization of the thermal expansion coefficient on the mass block significantly adjusted the preloading effect of the whole system [33]. Here, the coefficient of the thermal expansion of the mass block is optimized to 16.8 × 10^−6^ °C. The sensor has a good thermal impact response, and the system is stable. Figure 18 shows the dynamic responses of piezoelectric sheet with the mass block of its optimized coefficient of thermal expansion. The details of system responses are given in Table 4.

With the optimized coefficients of thermal expansion for mass block, the thermal–structural performance of the sensing system has been enhanced in the sense that the baselines of the measurement has fluctuated in a smaller range (11.5 MPa), and the sensing system can work adequately at all of the designated working temperatures, which means the high-temperature resistance capacity of the sensing accelerometer is much better than piezoelectric accelerometer 2271A from Endevco Corporation.

## 6. Conclusions

Existing approaches to design reliable accelerometers for intelligent weapon systems require many experiments to optimize the design of sensing systems. We propose a simulation-based design and optimization to reduce the development time and cost. Regarding the complexity of multiphysics behaviors and high level of uncertainty in the sensing acceleration of the weapon system, the proposed design methods include the following innovations.

(1) A prototype of the virtual experiment and its simulation technology are developed to reveal the couplings of thermal loads and impact loads over the sensing structure. Accordingly, the simulation model takes into accounts of all major factors such as preloads of bolts, temperature changes, and dynamic impact loads.
(2) A new form of quasi-static analysis method is proposed. It uses the result of dynamic analysis to express a static mode of the objects to be investigated. The method was adopted to represent the effect of initial preloads and thermal loads. The method has great potential to be extended to represent any static mode with a sequence of dynamic loading conditions.
(3) The simulation of the initial design has shown that the sensing system could only work below a temperature of 260 °C, and it led to the conclusion that the thermal expansion coefficient of the sensing system affected the performance of the sensing system significantly when subject to a high temperature.
(4) The simulation-based method was adopted to improve the sensing system by optimizing the coefficients of thermal expansions of the mass block. It led to an optimized sensing system, which can survive without a malfunction up to a temperature of 648 °C. This case study has shown the effectiveness of the proposed method for design optimization of accelerometers under extremely harsh conditions.

## Figures and Tables

**Table 1:** Geometric dimensions of parts in sensing system.

Part	Dimension Parameter	Dimensions (mm)				
Piezoelectric sheet	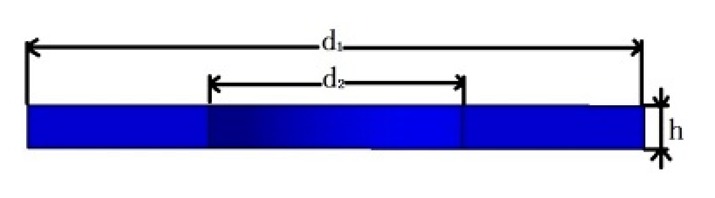	d_1_ = 10		d_2_ = 4.1	h = 0.6	
Conductive sheet	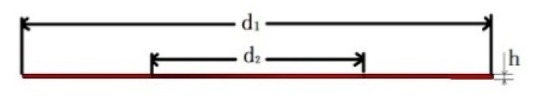	d_1_ = 10		d_2_ = 4.5	h = 0.2	
Insulating sheet	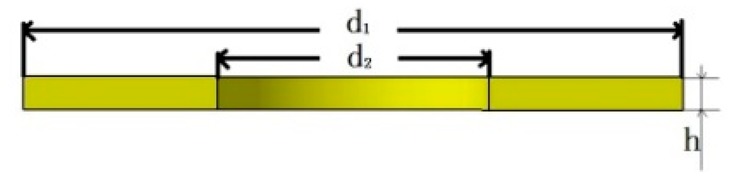	d_1_ = 10		d_2_ = 4.1	h = 0.6	
Mass block	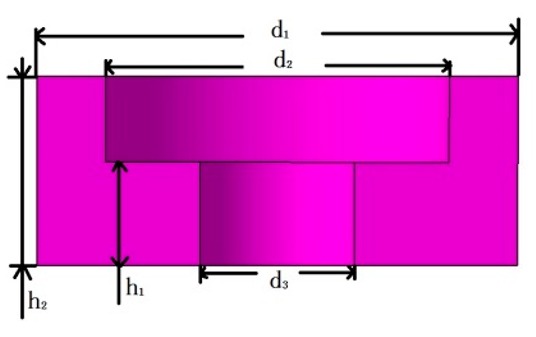	d_1_ = 14	d_2_ = 10	d_3_ = 4.5	h_1_ = 3	h_2_ = 5.5
Nut	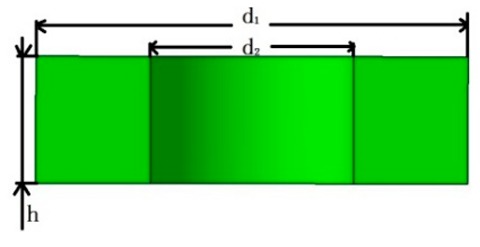	d_1_ = 8.5		d_2_ = 4	h = 2.5	

**Table 2:** Material properties of parts in sensing system.

Parts	Material	Density (g/mm³)	Elastic Modulus (Pa)	Poisson’s Ratio	Thermal Expansion Coefficient (1/°C)
Piezoelectric sheet	PZT5	7.77 × 10^−3^	2.10 × 10^11^	0.30	7.46 × 10^−6^
Center bolt	GH4093 alloy steel	8.19 × 10^−3^	2.26 × 10^11^	0.30	1.19 × 10^−5^
Conductive sheet	Cr20Ni80 alloy steel	8.40 × 10^−3^	1.80 × 10^11^	0.33	1.80 × 10^−5^
Insulating sheet	95 ceramic	3.70 × 10^−3^	2.21 × 10^11^	0.22	7.33 × 10^−6^
Mass block	Tungsten alloy	17.5 × 10^−3^	3.40 × 10^11^	0.27	6.75 × 10^−6^
Nut	GH4093 alloy steel	8.19 × 10^−3^	2.26 × 10^11^	0.30	1.19 × 10^−5^
Shell	GH4093 alloy steel	8.19 × 10^−3^	2.26 × 10^11^	0.30	1.19 × 10^−5^

**Table 3:** The responses of impact loads under different thermal conditions.

Temperature/°C	−70	−55	260	482	648
Action of initial bolt preloading/MPa	−59.1	−59.1	−59.1	−59.1	−59.1
Bolt preload coupled with thermal/MPa	−64.8	−63.4	−37.1	−14.8	0.0
Action of thermal/MPa	−5.7	−4.3	+22.0	+44.3	+59.1
Action of forward impact/MPa	−89.8	−88.5	−60.8	−39.9	−48.8
Action of inverse impact/MPa	−45.7	−43.8	−20.1	−4.1	0.0
Failure or not	Safe	Safe	Safe	Failure	Failure

**Table 4:** The details of system responses with optimized coefficients of thermal expansion.

Temperature/°C	−70	−55	260	482	648
Action of initial bolt preloading /MPa	−59.1	−59.1	−59.1	−59.1	−59.1
Bolt preload coupled with thermal/MPa	−59.3	−59.2	−57.1	−52.3	−47.8
Action of thermal/MPa	−0.2	−0.1	+2.0	+6.8	+11.3
Action of forward impact/MPa	−85.2	−84.5	−81.5	−77.5	−74.9
Action of inverse impact/MPa	−40.2	−41.1	−40.8	−34.9	−28.8
Failure or not	Safe	Safe	Safe	Safe	Safe
